# Developing Intra-EVA Science Support Team Practices for a Human Mission to Mars

**DOI:** 10.1089/ast.2018.1846

**Published:** 2019-03-06

**Authors:** S.J. Payler, Z. Mirmalek, S.S. Hughes, S.E. Kobs Nawotniak, A.L. Brady, A.H. Stevens, C.S. Cockell, D.S.S. Lim

**Affiliations:** ^1^ School of Physics and Astronomy, University of Edinburgh, Edinburgh, UK.; ^2^ Kennedy School of Government, Harvard University, Cambridge, Massachusetts, USA.; ^3^ BAER Institute, Moffett Field, California, USA.; ^4^ Department of Geosciences, Idaho State University, Pocatello, Idaho, USA.; ^5^ School of Geography and Earth Sciences, McMaster University, Hamilton, Canada.; ^6^ NASA Ames Research Center, Moffett Field, California, USA.

**Keywords:** Analog, Science operations, EVA, Mars, Workspace

## Abstract

During the BASALT research program, real (nonsimulated) geological and biological science was accomplished through a series of extravehicular activities (EVAs) under simulated Mars mission conditions. These EVAs were supported by a Mission Support Center (MSC) that included an on-site, colocated Science Support Team (SST). The SST was composed of scientists from a variety of disciplines and operations researchers who provided scientific and technical expertise to the crew while each EVA was being conducted (intra-EVA). SST management and organization developed under operational conditions that included Mars-like communication latencies, bandwidth constraints, and EVA plans that were infused with Mars analog field science objectives. This paper focuses on the SST workspace considerations such as science team roles, physical layout, communication interactions, operational techniques, and work support technology. Over the course of BASALT field deployments to Idaho and Hawai‘i, the SST team made several changes of note to increase both productivity and efficiency. For example, new roles were added for more effective management of technical discussions, and the layout of the SST workspace evolved multiple times during the deployments. SST members' reflexive adjustments resulted in a layout that prioritized face-to-face discussions over face-to-data displays, highlighting the importance of interpersonal communication during SST decision-making. In tandem with these workspace adjustments, a range of operational techniques were developed to help the SST manage discussions and information flow under time pressure.

## 1. Introduction

Not since the Apollo missions has human spaceflight involved exploratory science on the surface of an astronomical object. To prepare for the next phases of human spaceflight, the support architectures used for these missions must be updated to consider the technological and operational advancements made since Apollo. We will also have to contend with the communication challenges imposed by exploring Mars and other distant locations, which will likely include inherent bandwidth constraints and data transmission latencies (Love and Reagan, 2013; Beaton *et al.,* 2017).

Earth-based science teams will undoubtedly be vital as a supporting mission element for human missions to Mars and other deep space locations. Robotic missions to Mars have demonstrated the effectiveness of ground science team participation in daily planning and success outcomes (Squyres *et al.,* 2004; Squyres, 2005; Bass and Talley *et al.,* 2008; Smith *et al.,* 2008; Grotzinger *et al.,* 2012; Vasavada *et al.,* 2014). Science teams will participate in human space exploration across the various stages, including pre-mission planning (*e.g.,* site selection), intra-mission (*e.g.,* traverse planning, tactical and strategic), and post-mission (*e.g.,* data analysis, dissemination). One aspect of a science team's potential remit to astronauts operating on Mars would be to provide scientific guidance during extravehicular activities (EVAs), henceforth referred to as intra-EVA periods. It is important to note that how an Earth-based science support system will be implemented during a Mars human mission remains an open question. Scientists may have input on an inter- and/or intra-EVA basis, depending on which is judged to be most effective in the martian context.

Historically, a team dedicated to intra-EVA science support has been important to the success of human exploration. During the Apollo program (1961–1972), the Field Geology Experiment Support Room (Eppler, 2013), provided tactical (decisions made in the context of a single EVA) strategic (decisions made in the context of overall mission objectives) expertise to the crew during EVAs and ensured scientific information was rapidly disseminated out to the wider community (Connors *et al.,* 1994; Schaber, 2005; Clark, 2010). As a consequence of its success, several analog programs looking toward NASA's plans for a human mission to Mars (Drake *et al.,* 2010; Craig *et al.,* 2015) have attempted to integrate ground-based intra-EVA science support teams into their mission architectures. Desert Research and Technology Studies (DRATS) (Eppler, 2013; Gruener *et al.,* 2013; Ross *et al.,* 2013; Yingst *et al.,* 2013) and the Pavilion Lake Research Project (PLRP) both incorporated an intra-EVA science support team, the former working without communication latency. PLRP utilized a one-way light time (OWLT) communication latency of 5 min (10 min round-trip), which was found to strain the science team's ability to tactically acquire, process, and provide directions from the ground, particularly when the scientific objectives of the EVA were survey, representative in nature, as opposed to precise, instrument-data driven (Miller *et al.,* 2016). Such findings led to the concepts of operations (ConOps) design used at NASA Extreme Environment Mission Operations (NEEMO) (Chappell *et al.,* 2017) and Biologic Analog Science Associated with Lava Terrains (BASALT) (Beaton *et al.,* 2017), the latter being the setting for this study.

The objectives for this study were to examine and document the roles, responsibilities, strategic division of labor, and other operational techniques devised by a ground team that provided intra-EVA science support for real (nonsimulated) biological and geological science during simulated martian EVAs. These EVAs were conducted within the wider BASALT research program. Significant effort was put into achieving high-fidelity EVA simulations that allowed this study and others to make meaningful conclusions for future analog programs and real missions. The full test parameters, including rationales and justifications for the simulation test conditions, operational and scientific procedures, equipment and capabilities are covered in the works of Lim *et al.* (2019), Beaton *et al.* (2019a, 2019b), and Beaton *et al.* (2017). A truncated description of the overall test conditions is included in Sections 1.1 and 1.2 to provide context.

### 1.1. The BASALT research program

The BASALT research program examines geology and habitability in terrestrial volcanic terrains under simulated Mars mission constraints (Lim *et al.,* 2019).

BASALT consists of In-Field and Out-of-Field (X-Field) components. X-Field activities make up the majority of the yearly research, development, planning work. In-Field activities consist mainly of 3-week-long deployments involving the relocation of a significant portion of the BASALT team to a field site for 10 days of EVA simulations. The motivation for these EVAs was to test and exercise relevant operational and technological concepts in a simulated martian EVA that was conducting real science in a Mars analog environment.

The field sites used for these deployments were the eastern Snake River Plain Craters of the Moon National Monument and Preserve (COTM) in Idaho and Hawai‘i Volcanoes National Park, Mauna Ulu Region, Hawai‘i. These complementary sites were selected due to the presence of geological features analogous to modern and ancient Mars (see Hughes, *et al.,* 2019) which could be used to answer the BASALT team's core science question: How do microbial communities and habitability correlate with the physical and geochemical characteristics of chemically altered basalt environments?

### 1.2. EVA concepts of operations and support structures

BASALT deployments had 10 mission days with one nominally 4 h EVA per day. As mentioned, EVAs were carried out with OWLT communications delays of 5 or 15 min (which are within the 4–22 min OWLT delays experienced on Mars) and low (0.512 Mb/s uplink, 1.54 Mb/s downlink) and high (5.0 Mb/s uplink, 10.0 Mb/s downlink) bandwidth conditions (conservative upper and lower estimates for future Mars infrastructure) (see Beaton *et al.,* 2017). During these EVAs, two-person extravechicular (EV) teams were tasked with exploring, identifying sampling locations, deploying instrumentation, prioritizing, and ultimately collecting samples from the field sites. Each EV crew member was equipped with an extravehicular Informatics Backpack (EVIB), biological and geological sampling equipment, handheld scientific instrumentation (*e.g.,* X-ray fluorescence), and a wrist display (see Lim *et al.,* 2019). EV teams followed planned traverses to 10 m diameter target stations (typically 2–3 per EVA) selected to address science objectives in the months before deployments by the science team using satellite imagery.

Supporting the EV team over real-time communications were a two-person intravehicular (IV) team and through delayed communications a Mission Support Center (MSC). IV teams acted as the primary conduit of information between the MSC and EV crew, distilling science information, keeping track of timelines, and assisting in navigation. IV crews communicated nearly constantly with EV crews primarily though voice but could also send images and other data to the EV wrist displays (Lim *et al.,* 2019). The MSC housed BASALT's intra-EVA science team, herein referred to as the Science Support Team (SST), as well as other technical support and research groups working out of simulation. The SST communicated with IV using text, with no hard limits to the number of messages that could be sent. It is important to note that the SST did not have final decision on any aspect of the EVA and functioned only in an advisory role. Final decision rested with EV. See the works of Lim *et al.* (2019) and Beaton *et al.* (2019a, 2019b) for more details and representative figure of the overall simulation structure.

The operational concepts applied during the BASALT deployments were based on current mission architectures for Mars human exploration as well as best practices derived from other analog research program results (Lim *et al.,* 2011; Abercromby *et al.,* 2013; Chappell *et al.,* 2013; Eppler, 2013; Yingst *et al.,* 2013; Miller *et al.,* 2016) and were designed to allow the SST to tactically react to events during intra-EVA periods (Beaton *et al.,* 2019a). EVAs were divided into five distinct phases (with some representative times): (1) approach (1 h); (2) contextual survey (10 min); (3) sample location search (1 h) (2 and 3 merged in 2017 deployment); (4) presampling survey (1 h); and (5) sampling (50 min). Provided none of the predetermined flight rules (*e.g.,* hard stop times) were broken, EV crews could extend or shorten preplanned EV phases when required (Beaton *et al.,* 2017, 2019a and 2019b).

The approach phase was used by EV to traverse from the EVA start point to the first station. In the contextual phase, the station's regional context was described by the EV crew using voice and images, all of which were transferred to IV and MSC. Once the EV teams had performed their contextual descriptions, they began searching for potential sampling locations. This involved identifying locations believed to meet the EVA science objectives, placing markers close to them, and taking contextual and close-up still images. These images and descriptions were used by the SST to prioritize sampling locations for instrument deployment. The EV team then deployed field instrumentation (see Sehlke *et al.,* 2019) in the presampling survey at a subset of the proposed sample locations, guided by priority recommendations generated by the SST. Instrument data was then assessed by the SST together with the images and descriptions and a final recommended priority list returned to EV, who could begin sampling the top priorities (depending on the objectives). Typically, 2–3 sets of samples were collected, with a full set consisting of seven different samples, each collected using methods devised by the science teams during X-Field periods (see Beaton *et al.,* 2017).

A key aspect of this ConOps is that EV crew members completed the sample location search and the presampling survey at every station before moving on to the next phase; thus, they were required to return to sampling locations as they progressed through the phases, rather than completing all the phases at each location before moving on. This is important because it allowed the SST time to give tactical feedback on the earlier parts of each phase over communication latency. For example, the EV crew would deploy instruments at all sampling locations which might be located in multiple stations, before moving on to the sampling phase, allowing them time to get feedback from the SST (which was communicating over delay) on the instrument data from the first locations, before moving into the sampling phase. Although this method forced the SST to send initial priority lists before having a full set of information for all sample locations, it allowed it to meaningfully influence decisions without the EV/IV crew members having to idle while waiting for transmissions to be received over the Mars-like communication latencies.

The SST was composed of a set of Subject Area Experts ranging in experience level and scientific background but all acting in various science support roles for each EVA. During each EVA, the SST provided the IV/EV crew members with regular feedback and guidance regarding data collection methods, *in situ* analytical results, and operational timelines. For each of BASALT's 2016 deployments at COTM in Idaho and at Hawai‘i Volcanoes National Park, the MSC/SST to EV field crew members distance was approximately 50 and 15 km, respectively.

It is important to distinguish between the SST and general BASALT science team present at each deployment. The general BASALT science team describes the team guiding the science during deployments. Many of this team were assigned to the SST during EVAs, but some were part of other teams such as the Field Support Team. The SST also included engineers typically as the science communicator (SCICOM) who were technically not part of the deployment's science team. The SST as a discrete entity only really existed for approximately 1 h before the start of an EVA for preparation, to just after the EVA finished. Its members then reconvened for the Science Operations Working Group (SOWG) meeting and were joined by other non-SST members such as IV and/or other technical and management personnel.

To contribute to the development of Earth-based SSTs which will be vital to the support architectures of future Mars and deep space missions, we examine both how the SST set up their work space (how they reflexively adjusted work practices and the work space to better support receiving and discussing incoming data and their time-sensitive deliberations for science objectives and data collection) and the SST's roles and active management of information during EVAs. These aspects were examined over the course of two BASALT deployments to Idaho (June 2016) and Hawai‘i (2016). The Hawai‘i 2017 deployment will be covered in later papers that build on this work.

## 2. SST, Roles and Responsibilities

Here we describe the function and responsibilities of each SST role during BASALT's 2016 deployments, and the revisions made to improve SST function between Idaho and Hawai‘i. During these deployments, the SST was typically composed of 10–16 people, a number that fluctuated given the availability of personnel. The group was predominantly composed of scientists who developed BASALT's science objectives for each deployment and helped plan the EVAs, enabling them to become familiar with the science and operational aspects of each EVA. These scientists were distributed relatively evenly between senior, mid-career, and early career scientists, graduate students and undergraduate students. The majority of scientists had previous mission analog experience with programs such as DRATS, PLRP, and NEEMO, though some had none. The science group included geologists and biologists with expertise in volcanology, petrography, geochemistry, remote sensing, biogeochemistry, and astrobiology. Some scientists also had expertise utilizing scientific instruments, including spectrometers and remote sensing techniques.

The SST was required to provide the EV/IV crew members with quality information regarding field instrument deployment and geological and biological sampling over 5–15 min OWLT communication latency. This mainly included clear and well-thought-out instrument deployment and sampling priorities but also involved providing advice on sampling techniques and helping keep the EV crew aligned to the science goals.

During the EVAs, the main task of the SST was to interpret and discuss observational and instrumental results being sent by the EV crew in order to identify sampling targets that would meet the predetermined strategic scientific objectives (Brady *et al.,* 2019). The SST had to achieve this under tight time constraints with sometimes only a few minutes to debate a sample location, given only limited scientific data such as still images, handheld field spectrometer readouts, voice communications, and video access that changed given bandwidth conditions (see Beaton *et al.,* 2017; Sehlke *et al.,* 2019; Stevens *et al.,* 2019). To manage these requirements, a range of SST roles were established to provide crucial personnel with appropriate information, facilitate scientific discussion, enable collaborative decision-making, and preserve a command structure. A description of each position and the reason for its integration into the SST are detailed below.

### 2.1. SST positions

The roles listed below were part of a hierarchical structure that gave primary communication control to a primary communicator between the SST and IV/EV.

#### 2.1.1. Science Support Team Lead

Discussion and debate among the SST's specialists were critical to its function. However, scientific debate may lead to protracted disagreements, which can be problematic under the time constraints. During the time constraints of EVAs, it was crucial to manage the complexity and variety of information being leveraged in the SST and the range of different personnel with varying expertise. As such, it was necessary to have a command structure in place to ensure timely discussions and effective information management. To achieve this, a SST Lead was utilized during all EVAs at both BASALT field deployments.

The SST Lead position was typically held by a senior or mid-career scientist with at least some analog experience. The SST Lead was responsible for managing SST communication during EVAs. It is important to note the SST Lead was not responsible for the overall mission science objectives. These were devised by the wider BASALT science team during X-Field periods. During pre-EVA preparation, the SST Lead managed workspace setup, telecommunication and software systems checks, and personnel briefings. During the EVA, the SST Lead was understood to be in charge of managing group interactions, directing attention to tasks and timelines, and providing the final SST decisions to IV/EV teams awaiting feedback or directions in collaboration with the SCICOM. This role included facilitating, and sometimes prompting, debate among the group, ensuring each member performed their role effectively, soliciting decisions on sampling priorities, calling consensus votes if required, and coordinating with the Simulation Coordinator (SIMCORD) on SST-related events. Post EVA, the SST Lead led the SOWG meeting attended by most of the SST and other scientists involved in the sim, and later represented the SST during a Mission Management Team meeting held at the end of each day.

#### 2.1.2. Science communicator (SCICOM)

The SCICOM position was created to monitor operational concerns in order for the rest of the SST to focus on scientific deliberation and decision-making. They were also ultimately responsible for communications between SST and EV, although they worked loosely with the SST Lead on this. A key aspect of the SCICOM role was to ensure that sample priority input was transmitted to the IV prior to deadlines imposed by the EVA ConOps. For this reason, the position was typically filled by an engineer with an operational research background. Because BASALT simulated communication latency and employed IV positions, SCICOM communicated with IV, not EV. Communication with IV was done via text using Playbook's mission log (part of the Minerva software suite which enables timeline management and creation, traverse planning, data collection and archiving, and procedure management [Marquez *et al.,* 2013, 2019]). Delayed text communication with IV meant that some aspects of the SCICOM role used in an analog like DRATS, where communication was real-time through voice (Eppler, 2013), were necessarily transferred to the IV team.

SCICOM, in close coordination with the SST Lead, was thus responsible for communicating SST decisions to space and informing the SST Lead of any operational concerns. SCICOM closely tracked EVA phases, timelines, upcoming deadlines, and other relevant operational factors to ensure that SST input was provided to the IV/EV crew prior to the start of the next phase of the EVA. During BASALT, the Minerva software suite supported these tasks (Marquez *et al.,* 2019). SCICOM worked closely with the SST Lead to compose messages to IV/EV, which included critical instrument and sampling priority messages and associated scientific rationale. SCICOM's role in this task was to ensure the operational content of messages was sound, while the SST Lead managed its science content. Due to the complexities of instrument deployment and sample collection, the structure and content of messages from ground to space were found to be particularly important to the success of the EVA. Message composition is discussed in more detail in the work of Kobs Nawotniak *et al.* (2019).

#### 2.1.3. Specialists: Science Tactical

Among the SST, typically two to three scientists were given role of Science Tactical and were responsible for providing various areas of scientific expertise to deliberations and discussing instrument deployment and sampling priorities. Science Tactical made recommendations for best possible sampling through the use of images, field spectrometer readouts, voice communications and video from the EVA teams. The leaderboard (for details, see Stevens *et al.,* 2019), a dynamic representation of these recommendations on a shared display, was updated during deliberations until the Science Lead called for a final vote, at which time Science Tactical gave their final recommendation for sampling priorities. In this way, Science Tactical deliberations were ultimately used to ensure EV sampled the best possible rock to satisfy the EVA science objectives.

All Science Tactical personnel freely communicated with other SST members during EVAs, which enabled interdisciplinary interactions for problem solving. Access to data collected during an EVA occurred through the Exploration Ground Data System (xGDS) (see Deans *et al.,* 2011), a part of the Minerva software suite (Deans *et al.,* 2017; Marquez *et al.,* 2019), a core supporting capability for the BASALT missions.

#### 2.1.4. Specialists: Remote Science Tactical

A small number of Science Tactical specialists participated in both 2016 BASALT deployments remotely from locations around the world. These specialists had access to the delayed audio feed through a virtual multi-channel, multi-intercom system (VCOM™), and delayed EV and Situational Awareness (SA) video, still images and notes through xGDS. It was possible for them to communicate via direct messaging with individual SST members who were located in the MSCs during EVAs and to contribute to identifying features of interest on the video and audio feeds. The rest of the SST could see their notes in xGDS and act on them more directly if required. As distributed science teams were not part of the BASALT ConOps design, Remote Science Tactical specialists did not receive additional tools that would enable improved integration with the rest of the SST. Further study is required to determine how best to implement fully or partially distributed SST teams in the future.

#### 2.1.5. Leaderboard Lead

During EVAs, information and data from IV and EV flows rapidly into the SST. The SST must review incoming material, deliberate, and reach a consensus within prescribed time durations. Keeping track of incoming data (*e.g.,* sample locations), suggested sampling priorities, and final consensus decisions was difficult during deliberations. To alleviate this issue, the Leaderboard Lead was tasked with managing the dynamic leaderboards: tables that recorded and displayed SST sample location preferences on a moment-by-moment basis. Three were typically used during an EVA. One, the master leaderboard, was used by the Leaderboard Lead to manage SST priorities and was not displayed to the rest of the SST. The second and third were simplified versions showing priorities for the presampling survey and sampling phases of the EVA, and were displayed to the SST during these phases (for figures and more information on the leaderboards, see Stevens *et al.,* 2019). Since the master leaderboard could become dense with information and displaying priority information from previous phases sometimes led to confusion, multiple leaderboards were used to make it easier for the SST to understand priorities at a glance.

#### 2.1.6. Still Image Management

The role of Still Image Management was responsible for handling a high volume of incoming still images that arrived to the SST and were made available for viewing via xGDS. While the SST found still images to be critically important for examining potential sampling and instrument deployment locations, the images arrived without contextual information or labels that would facilitate ease of use. The SST had to listen for this information, which could be heard on the dedicated audio channel (VCOM). Another method they used was to locate the timing of the image relative to the EVA timeline. This method was slow and time consuming. Additionally, occasional network instabilities meant that image arrival could be sporadic and, given the busy SST schedule, often missed, contributing to the SST falling behind in location assessment. The Still Image Management role primarily focused on tracking and orally announcing image arrival and attaching key information such as sample and context descriptions and sample locations to images in xGDS.

#### 2.1.7. Specialist Leads

Specialist Lead roles were created to aid in focusing the science conversation by identifying SST members with specific knowledge that established them as topical experts. One Specialist Lead was assigned for each of the three tactical specialist categories: geology, biology, and instrumentation. As Subject Area Experts, they led discussion specific to their tactical themes and ensured that relevant information was properly represented in the final decisions. These leads were particularly useful when SST discussion fragmented into several specialist debates and therefore required a person to manage and inform the SST Lead of the outcome. They also assisted the SST Lead in managing the overall SST conversation, particularly when the SST Lead was taken away from the general science conversation by other tasks. This position was first employed during the Hawai‘i 2016 deployment (see Section 2.2).

#### 2.1.8. Situational Awareness (SA)

Situational Awareness was tasked with constantly monitoring voice communications (through a headset) to track the EV team's progress.^1^ While these individuals did not enter discussions on science points, they were available upon request to update the SST on the EVA team's current activities and position during the EVA. Nominally there were two SA personnel, such that one could continue to monitor the audio and video feeds while the other reported updates to the SST. This position was also first employed during the Hawai‘i 2016 deployment (see Section 2.2).

### 2.2. Lessons learned

A key lesson learned from the SST during the COTM deployment was the benefit of having backups to certain tasks. During quieter parts of the EVA, SST members were typically able to balance multiple tasks. However, that became very difficult once complex scientific deliberation started, particularly during faster-moving EVAs. Consequently, having built-in mechanisms to pick up the tasks necessarily left by others prevented the team from becoming disorganized. This manifested as creating the Specialist Leads and SA positions for the 2016 Hawai‘i deployment.

The Specialist Lead position addressed a problem where the Science Lead would periodically be removed from the science conversation when working on drafting and sending messages to IV with SCICOM. This was particularly apparent during faster-moving EVAs (under the 5 min OWLT communications latency). To manage this, the Science Lead would designate either the Biology or Geology Specialist Lead as a second in command to provide leadership and ensure the deliberations remained on track whenever the Science Lead was otherwise occupied.

Similarly, the SA role addressed an issue where SST members struggled to keep track of the EVA teams spatially in the field and temporally within the timeline as they moved into phases requiring active discussions of incoming data and sample location prioritization. While Playbook was used to track the EV team's location in the general phases of the EVA, it did not include information on the individual components of a particular phase (*e.g.,* EV team is sampling site AB). The SA role was used to solve this problem by dedicating personnel to the role.

The addition of these two positions in Hawai‘i 2016 resulted in perceived improvements in organization and smoothness of SST operations and highlighted the importance of identifying tasks likely to benefit from built-in redundancy in a time-pressured environment with fluctuating workloads.

The SA position does not appear to have been used in previous analog programs. This may be due to several factors, including the diversity of expertise employed at BASALT (geology, biological science with instrument use), the number of personnel in the SST, and perhaps most importantly the presence of complex time-limited debate occurring simultaneously with a fast-moving EV crew. It is likely during a real Mars mission, positions like SA, or a technological substitute, will be an important part of the SST.

Science Lead/IV/EV/SCICOM positions were rotated among six people during the Hawai‘i 2016 BASALT deployment. Personnel rotation allowed the crew to better appreciate different role challenges and limitations and improved awareness of how the positions interlink. Prior to the implementation of personnel rotation, EV/IV crew reported difficulty in understanding how content in the transmitted audio feed could be missed in the SST; after rotation, the same crew members not only stated that they better understood the limitations associated with consensus-building discussion, but they also adapted their own actions in EV and IV roles to compensate for those challenges.

Successful communication and information management among 10–16 SST members were facilitated in part by the size of the workspace in Hawai‘i. There was enough room to reconfigure seating in response to number of participants and to support individual communication differences. It is the authors' intention that the number range and room configurations described here will benefit future studies on ideal team and work-space sizes for colocated SSTs.

## 3. SST Layouts

The built environment for any team can support or interfere with communication, information flow, contextual awareness, and concentration. During EVAs, the SST team's physical proximity to one another and the information arriving from the field affected the time-sensitive activity data management, review, and deliberation. SST development for human missions to Mars and other deep space locations is an underdeveloped area. Toward building these case studies, we offer details on the workspace configurations and some usage descriptions for BASALT 2016 deployments noting good practices, challenges, and workarounds.

For each of BASALT's 2016 deployments, the MSC and SST physical workspace varied in size and layout. At each site, the MSC was housed in unalterable buildings (*i.e.,* walls and doors could not be moved). However, within the MSC there was limited flexibility for rearranging workspace components such as chairs, people, tables, A/V equipment, and individual and shared monitors. Across deployments, the SST regularly assessed communication and workspace issues in post-EVA meetings.

Considering workspace configurations, including the placement of people, seating, monitors and other tools, can yield arrangement schemas that better support communication and information flow (Roger and Agarwala-Rogers, 1976; Suchman, 1987; Hatch, 1990; Harper *et al.,* 1991; Tompkins, 1993). The study of workplace ergonomics identifies the importance of tracking human-technology interactions to inform questions on the types of materials that should be used or need to be developed in order to support the particular work being conducted (Pheasant, 2016). High-tech work settings, where digital mediums play a significant role in the communication process (*e.g.,* projectors, monitors, laptops) and in obtaining information (*e.g.,* field instruments used to collect chemical data), necessarily increase the number of communication relationships that are part of ongoing activities (Wenger *et al.,* 2009). Studying BASALT's science team workspaces and the team's reflexive adjustments can contribute to developing normative workspace configurations that best support collaborative multidisciplinary science team (colocated and remote) decision-making.

### 3.1. Idaho 2016

**FIG. 1.**
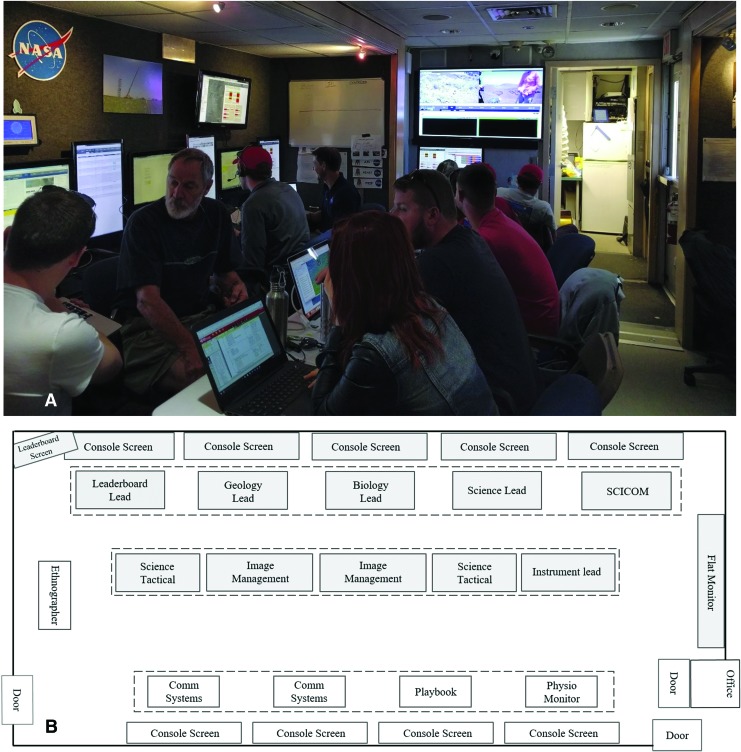
(**A**) BASALT Idaho 2016 SST workspace. (**B**) SST workspace layout and seating arrangement for 10 SST roles. There was space for two rows of seating for the SST, facing one side of the MSC trailer. Other BASALT team members (non-SST roles in white boxes) occupied a table console and seating that faced the opposite side of the MSC. Photo credit: Zara Mirmalek.

During Idaho 2016, the SST workspace occupied two-thirds of the MSC, a recommissioned windowless mobile trailer 16 m (53 ft) long by 2.5 m (8.5 ft) wide, with two sections extendable to produce a width closer to 4.5 m. The SST was set up in one of these extended-width sections. The remaining one-third of the MSC was occupied by other BASALT workgroups including the communications, physiology, Minerva software development teams, and the IV workstation. The absence of windows in the MSC helped separate the team from the local natural environment and focus on the remote location visible via video and imagined via audio (Fig. 1). The SST personnel were seated in two rows (Fig. 1). In the first row, five SST persons sat at consoles spanning the length of the SST workspace. In the second row, four to five SST persons sat at a long table facing the chair backs of row one. Spacing between seats was minimal, and the spacing between rows was narrow, approximately the width of a standard office chair. These tight quarters made it challenging for scientists in the first row to stand (as needed for some communication acts such as calling for attention or speaking to someone two seats away) without disrupting their neighbors.

Each SST person had sight lines to the shared displays (*e.g.,* projector screen and the aforementioned leaderboard monitor), showing critical BASALT field operations information. The projector screen was located on a wall perpendicular to the SST's two rows (Fig. 1). The limited number of shared displays available to the team was conducive to focusing their attention to a limited number of shared reference points during SST discussions. The use of laptops (each person used one, some used two) gave people some flexibility to move seating positions should the switching of personnel in certain roles be required.

Despite the close proximity of rows one and two, there were times when it was difficult for SST members to hear one another, between and along rows. Some SST persons often took to rotating their seats, so they were facing the space between the two rows, allowing them to communicate with more of the SST. Such communication issues are common in the early development stages of a workflow, wherein a multidisciplinary team with varying levels of familiarity with one another begins working on turn-taking, managing information flow, and interpersonal communication habits. With its limited size and egress points, the constraints of the MSC also contributed to early communication issues. Seat shuffling, competition for power outlets, and foot traffic, including observers from the media, all created challenges for efficient and accurate communication in the SST as a result of the closely packed space.

### 3.2. Hawai‘i 2016

For BASALT Hawai‘i 2016, the MSC was a relatively large room, 15 m long × 7.5 m wide × 5 m tall (approximately), with four sets of windowed doors (1.6 m wide × 2.3 m high, approx.) along one wall. The windowed doors consisted of two adjoining doors that had glass panes from top to bottom, covered by a curtain which blocked out much of the external view. The SST workspace was set up on one side (about one-third) of the room (approx. 6.8 m ×7.5 m).

**FIG. 2.**
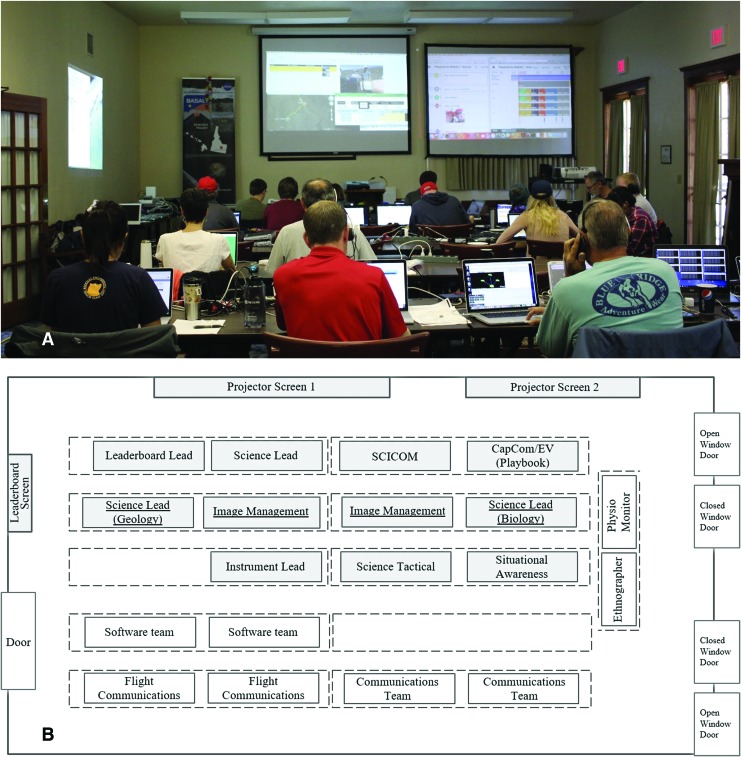
(**A**) BASALT Hawai‘i 2016 SST workspace. (**B**) Initial SST workspace layout and seating arrangement for 12 SST roles. There were three rows of seating for the SST, all facing toward the projector screens. Other BASALT team members (non-SST roles in white boxes) occupied a table to the side of the SST and the rows behind. Photo credit: Zara Mirmalek.

The SST's initial seating arrangement was similar to that of Idaho 2016, but the layout included three rows instead of two (Fig. 2). Each row seated about four SST members, a number that was partially determined by the large size of the chairs. In contrast to Idaho's standard office chairs and folding chairs, the chairs were heavy and wooden framed, with armrests and without wheels or swivel ability (chair dimensions: base height 47 cm, back height 83 cm, width 57 cm, depth 42 cm).

All SST seating faced a front wall on which hung two large (approx. 8 ft × 8 ft) shared projector display screens. Projected on these displays were EV and SA camera video, EV positional tracking via Google Earth, Playbook mission log and timeline, and Mars and Earth time displays. A third smaller screen, used for the SST leaderboard, was located on the left wall (the windowed doors lined the right wall; see Fig. 2). An additional row set up perpendicular to the three rows served BASALT researchers who were not a part of the SST: ethnographer and physiology monitor. The two seats in this row faced the left wall, where the leaderboard was displayed.

**FIG. 3.**
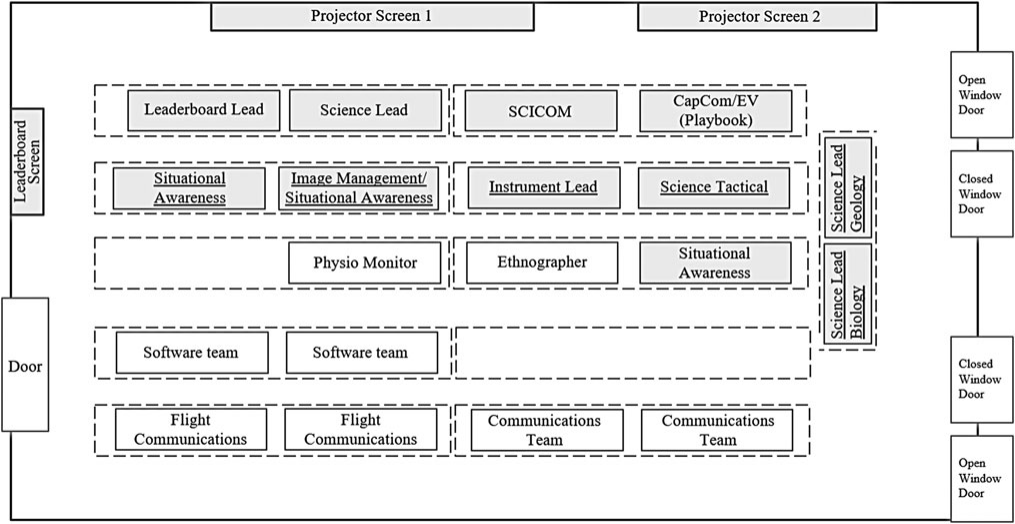
The SST's second seating arrangement during BASALT 2016 Hawai‘i deployment, reconfigured in response to workflow and communication needs. The larger size of the MSC compared to Idaho and ability to more easily move equipment gave team members the space to make changes to the SST's workspace. One key change was making the adjacent row part of the SST team seating and moving two Specialist Leads from the third row over to the fourth row. This new configuration suggested an oval-shaped seating arrangement, in contrast to linear rowed pyramid (see Fig. 2), and better supported face-to-face discussions.

Over the course of the Hawai‘i 2016 deployment, the SST changed the seating arrangement to better support their workflow (see Figs. 2 and 3, the roles underlined highlight those that changed).

Initially, the seating arrangement followed a traditional organizational hierarchy, which places the person with the most decision-making authority at the top, and each subsequent level below is populated with people with decreasing decision-making authority (Hatch and Cunliffe, 1997). The Science Lead and SCICOM were placed in the first row closest to the two projector screens. The Leaderboard Lead was also situated on this front row to communicate with the Science Lead regarding priorities and for their computer connection to the leaderboard projector. Rows two and three included the Specialist Leads, Situational Awareness, and Still Image Management personnel.

One of the problems with the three rows of seating was that people could not hear one another between rows one and three. During the practice EVAs that preceded the first full mission day, SST members explicitly acknowledged that people in row three could not be heard in row one. In response, the SST members in row one began asking row three for their participation as a matter of protocol. This initiative improved communication across rows; however, as the deployment progressed, communicating across rows one and three remained difficult.

Another issue with the initial seating arrangement was that the direction of the seating (all facing the front wall) placed the importance of the projector screen information over the importance of the face-to-face discussions among the SST. While data was streaming on the projector screens, the SST members in the first row found their attention directed toward the screens and in the opposite direction from teammates in rows two and three. The Science Lead often managed this problem by turning to face rows two and three. While this was effective, it did create an interruption in the shared view; that is, the Science Lead no longer shared the same constant view with the rest of the SST of information on the two projector screens. To maintain a connection to the EV video, GPS tracking, and Playbook mission log while facing the SST, one Science Lead held their laptop in their lap (rather than its location on the row one table) and used it to display the same content as was on the large projection screens. This practice was adopted by another Science Lead later in the deployment. While this improved the ability of the Science Lead to work with the SST at large, it was described as a physically uncomfortable workaround.

The most significant change to the SST workspace during Hawai‘i 2016 was the reconfiguration of the role locations (seating arrangements). To support face-to-face communication, seating was shifted so that people were physically positioned to look at one another without losing sightlines to the shared information displays (see Fig. 3). During deliberation, Specialists Leads needed to have face-to-face discussion with SST members in all rows, and these conversations involved verbal, gestural, and facial communication. In the initial setup, Specialist Leads were located toward the front of the SST, facing away from some positions, which led to disjointed communication. The SST decided to relocate the Specialist Leads from row two to the previously non-ancillary row perpendicular to rows one and two. SST members were then moved from rows three and four up to row two, which helped close the communication distances among them. In this way, the SST reconfigured their workspace from a pyramid that prioritized data displays (Fig. 2) to a long ellipse that prioritized face-to-face discussion (Fig. 3). One scientist's reflection on this change, from the pyramid setup that prioritized viewing the data displays to the long oval, was that “there is no point in having a team of experts in different disciplines if they can't all talk to each other and arrive at an information consensus.” Another scientist added, “The various SST personnel collectively decided to compress into the smaller physical space even when that meant barely having space on tables for their laptops. The team chose improved communication and group orientation over physical comfort for multi-hour EVAs.”

The development of workflows that respond to the needs of a multidisciplinary science discussion was shown to benefit from providing those scientists with a workspace that they could rearrange, as needed. During Hawai‘i 2016, the SST's changes to their workspace were possible because of the non-stationary seating, and the supply of mobile individual data displays (laptops) and power strips. An additional benefit to the spacious size of the MSC in Hawai‘i was that it allowed other BASALT workgroups developing SST-related work support technologies (*e.g.,* databases management, software, user interface) to work near and provide technical assistance to the SST without disrupting the workspace.

## 4. Operational Techniques

Through the 2016 BASALT deployments, several lessons were learned about how to organize and operate the SST during an analog EVA. As detailed above, these operational techniques were developed under a specific SST workspace configuration and BASALT ConOps but offer insights applicable to future analog programs and deep space missions.

### 4.1. Prior to EVA start

Science Support Team activities typically began approximately 1 h before the EVA start time. Pre-EVA activities began with a “roll call.” One by one, each SST member briefly described their EVA role and responsibilities, allowing the team to familiarize themselves with each role and ensure each SST member understood their own. Any uncertainties were solicited from the SST regarding the upcoming EVA, with members of the strategic planning team who prepared the mission day briefs providing clarification as needed.

Pre-EVA preparatory time was also spent doing systems checks to ensure the whole group had access to all the relevant informatics tools they individually required, including traverse plans and precursor data. Projector screen content such as the leaderboard, video feeds, and EV location on satellite imagery was configured and adjusted during this period.

### 4.2. EVA station approach and contextual survey

Each EVA started with the station approach and contextual survey phases, which gave EV the opportunity to examine the area around the prospective sampling locations, providing context to the SST (for EVA phase details, see Lim *et al.,* 2019 and Beaton *et al.,* 2017). During this period, the majority of information transmitted to the SST from EV consisted of visual descriptions and still images of the landscape around the stations and the stations themselves. Visual descriptions and still images became very important data collection tools in later EVA phases when selecting sampling locations. Therefore, this early phase was used by the SST to provide feedback to EV on the content of the verbal descriptions and still image composition. This helped ensure high-quality data was being collected by EV and received in the SST (for more details on SST-IV communication see Kobs Nawotniak *et al.,* 2019). During a real Mars mission this might have been less necessary due to more extensive EV training. The SST spent most of the approach and contextual survey phase recording pertinent information and discussing EV's observations.

### 4.3. EVA sample location search, presampling survey, and sampling

The sample location search and presampling survey (instrument deployment) phases were the busiest periods of the EVA for the SST. In these phases, the SST had to work cooperatively to assess each sample location identified by the EV team, rank the candidates for sampling on the dynamic leaderboard (see Stevens *et al.,* 2019), and communicate these rankings and justifications to the IV (see Kobs Nawotniak *et al.,* 2019). During the sample location search phase, the SST examined still images, listened to verbal descriptions from EV, and discussed which sample locations should be interrogated with field spectrometers. In the presampling survey phase, the SST assessed the results from the instruments to identify which of the proposed samples best met the EVA objectives such that those could be prioritized for final sampling.

The pace at which data entered the SST during these phases was often rapid. For the SST to keep up with the EV team, it was critically important to reorder and update the priority list on the leaderboard every time a new sample location was available for assessment. Allowing a backlog to build up during this phase always resulted in increased confusion, rushed discussion, and amplified stress. This was particularly pronounced during the lower latency (5 min OWLT) condition due to the SST's enhanced ability to interact with EV on time-dependent tasks, which increased workload. Occasionally the cadence of incoming data made keeping pace very difficult, particularly during early EVAs as the SST learned how to communicate efficiently. As a rule, the EV teams were never told to pause and wait for a SST decision, emulating the operational unacceptability of crew deadtime during an EVA.

The Science Lead was responsible for ensuring that the debate pace matched the EV team's progress through techniques such as requesting consensus votes on sampling priorities by show of hands. Timers (digital countdown timers) were also given to SST members and times called out to help individuals self-manage debate around impending deadlines. The Science Lead spent the majority of time during this phase leading discussion and soliciting opinions. Periodically, these responsibilities were transferred to the Specialist Leads to allow the SST Lead to work on constructing priority messages with SCICOM. In comparison to the 15 min OWLT condition, the 5 min OWLT increased the SST's ability to influence the EVA (see Stevens *et al.,* 2019). This meant the SST Lead was more prone to separating him or herself from the wider science conversation due to an increased number of messages being sent, requiring the Specialist Leads to step in more often to lead the science conversation. It also placed a greater time pressure on general conversations during this phase due to the additional options to consider, making them faster paced. Specialists required a short period of time, generally 1–5 min, to debate a new candidate sample before the Science Lead requested a priority order. Ideally, this happened a few minutes after all the data for a candidate sample in a particular phase had been received by the SST. Hurrying debate prematurely was always found to be distracting.

Once the EVA reached the sampling phase, the majority of the decisions requiring SST input had been made. The SST did have some input during this phase, including advising on sampling techniques, although this was diminished in the longer latency condition since feedback could not reach EV within actionable time. This phase was used by some of the SST to begin preparing debriefs on the current EVA and strategic planning or traverse mapping in Surface Exploration Traverse Analysis and Navigation Tool (SEXTANT) (Gilkey *et al.,* 2011) for the following day's EVA. After the EVA was complete, most of the SST members rejoined the larger science team for debriefings; others worked on instrumentation or capabilities.

## 5. SST Lead Variability

As mentioned, the Science Lead/IV/EV/SCICOM positions were rotated among six people during the Hawai‘i 2016 BASALT deployment. During the COTM and Hawai‘i deployments, the Science Lead position was rotated between four BASALT scientists. The tactics employed by each SST Lead to fulfill the position (outlined in Section 2.1.1) varied depending on mission context, personality, and experience. The Science Lead role requires more focus on management than on scientific data analysis and decision-making. Leveraging information from all SST members was important. As with any group conversation, SST deliberations could become dominated by small groups of more talkative individuals if not managed properly. The Science Lead often needed to actively solicit comments from various people whose voices were not as loud or who had not yet become familiar with the norms of colocated and time-sensitive science team deliberation. Overall, the team responded positively to the Science Leads' communication directions.

Science Support Team Leads benefited from sharing leadership responsibilities with the Specialist Leads. Given the amount of Science Lead responsibilities, a failure to work in partnership with the Specialist Leads sometimes led to the SST Lead becoming overwhelmed with responsibilities, some of which went neglected. The SST Lead working in partnerships with the Specialist Leads most often resulted in more robust discussions. Similarly, successful task management by the SST Lead also depended on their working relationship with the SCICOM; since these two personnel typically stayed as a partnership throughout their position rotations, each SST Lead–SCICOM pair developed their own style of load balancing and communication.

## 6. Future Scope

One area of interest for further study is the SST layout with a focus on the relationship between workplace technologies and ideal work support for the SST. It became clear from the Idaho and Hawai‘i deployments that the SST seat positioning was important to their productivity. A concentrated effort to determine the ideal SST layout, or a selection of ideal SST layouts related to the parameters of mission operations or analog, would be beneficial. This might involve placing data displays in novel positions to better balance the SST need to prioritize discussion while retaining their ability to maintain a shared view of large data displays. Additionally, assessment on the ideal locations for data and mission operations information across SST computer monitors and screen projectors could be achieved by studying the intersection of shifting of content across displays and SST communication in accordance with the latency variations.

Assuming a real future Mars mission SST will be larger and with a broader range of experts than present here (given the resources that would be available), adding more specialists to the SST and diversifying the expertise present should improve understanding on roles, configuration, and workspace layout. From this, the practicalities of team size used during time-pressured debates could be examined further. For example, scientists are often trained to operate relatively independently, each contributing their own efforts to a joint outcome. Rarely in terrestrial field science do researchers engage in continuous debate and consensus building throughout an entire project; it is even more unusual for such discussions to occur under extreme time pressure. As such, an excellent scientist and researcher may not necessarily be well suited to the SST environment. Successful SST members were confident in their own specialty areas but were also skilled at succinct and clear communication across a team of varying expertise. They needed to multitask throughout the EVA, for instance contributing to tactical science discussions while also managing the leaderboard. Importantly, SST members needed to be skilled listeners, learning from their teammates and developing a shared vocabulary that facilitated shared communication and task management.

Work on the integration of remotely located SST members could also be greatly expanded on. If a human mission to Mars utilizes a distributed SST design with a significant portion of its members working off-site, substantial advances would need to be made to allow them to operate effectively with the rest of the SST at the rapid pace of an EVA. This is a significant area of work and warrants further examination.

Testing SST function under other ConOps would alter the types of information entering the SST and modify the timing of discussion deadlines. Many of the lessons learned here related to organizing and running a SST would be applicable to new EVA structures, but these new techniques would bring their own challenges. Over time, testing a broader range of ConOps will result in the best study of SST techniques and guide the structure eventually used on a Mars mission or other deep space missions.

## 7. Conclusion

Through utilizing a SST during the two 2016 BASALT deployments, a range of lessons were learned about how to organize and effectively manage a SST during intra-EVA operations. The initial SST roles established in Idaho were improved upon during the Hawai‘i deployment by introducing Specialist Leads. This resulted in more effective management of technical discussions while providing a support structure for the Science Lead. The addition of a SA Lead allowed the specialist teams to concentrate on discussing science, freeing them from trying to track the EV team.

Providing SST members the space and equipment to rearrange the workspace to suit their needs was shown to be beneficial to their ability to cope with the rapid work rate during an EVA. This freedom resulted in movement away from a pyramid-shaped layout to something close to an oval, allowing the team their preferred option of prioritizing discussion over access to data displays. This preference represents a potential best practice that will be further tested during the 2017 BASALT deployment.

Practicing a rigorous preparatory phase before each EVA that included all SST members and EV/IV teams was essential in ensuring EVA success and minimizing participant frustration over miscommunication. Feedback between the SST and IV/EV in the quieter periods of the EVA allowed for iterative improvements to be made during an EVA, improving the quality of the data received in the SST. During the busiest phases of the EVA (sample location search and presampling survey), ensuring the SST kept on top of prioritizing sample locations as they became available was critical, with a backlog observed to cause numerous issues.

In the future, the SST will be a crucially important part of mission support as space exploration moves beyond low Earth orbit. SSTs of the future will be populated by cooperative, and sometimes competing, scientific interests; as such, it is critical to use analog simulations driven by interdisciplinary science to identify best practices for successful SSTs. Future work should investigate varying ConOps, SST sizes, human interaction, room design, and distributed teams, as these will all influence the SST experience. The lessons learned from these studies will be vital for improving the scientific return of high-risk and expensive human spaceflight missions to Mars and deep space.

## Footnotes

^1^ In this context, the phrase “situational awareness” refers to local environmental information needed by a person who is “remotely” present but physically distant, *i.e.,* a scientist working remotely from the physical field of study. It is part of the developing lexicon for scientists working in remote environments. Formalization of said lexicon will certainly address similarities and differences with “situation awareness” (and “situational awareness”) produced and used in aviation (Sarter and Woods, 1991).

## Abbreviations Used

BASALT: Biologic Analog Science Associated with Lava Terrains
ConOps: concepts of operations
COTM: Craters of the Moon National Monument and Preserve
DRATS: Desert Research and Technology Studies
EV: extravehicular
EVAs: extravehicular activities
IV: intravehicular
MSC: Mission Support Center
NEEMO: NASA Extreme Environment Mission Operations
OWLT: one-way light time
PLRP: Pavilion Lake Research Project
SA: Situational Awareness
SCICOM: science communicator
SOWG: Science Operations Working Group
SST: Science Support Team
VCOM: virtual multi-channel, multi-intercom system
X-Field: Out-of-Field
xGDS: Exploration Ground Data System
